# Reconstruction of severe acetabular defects (Paprosky type III A) in total hip arthroplasty using modular tantalum augments in combination with a cemented cup

**DOI:** 10.1007/s00264-024-06334-x

**Published:** 2024-09-27

**Authors:** David Spranz, Lisa-Marie Müller, Raphael Trefzer, Pit Hetto, Timo Nees, Tobias Renkawitz, Tilman Walker, Tobias Reiner

**Affiliations:** Department of Orthopaedics, University Hospital Heidelberg, Schlierbacher Landstraße 200a, 69118 Heidelberg, Germany

**Keywords:** Modularity, Total hip arthroplasty, Acetabular defect reconstruction, Tantalum trabecular metal augments, Hip revision arthroplasty

## Abstract

**Purpose:**

Acetabular defect reconstruction can be a complex and challenging surgical procedure, with stable long-term fixation of the implants remaining the ultimate goal. The purpose of this study was (1) to evaluate the radiological and clinical outcome of complex acetabular reconstruction surgery with the use of modular tantalum TM augments in combination with cemented revision cups; (2) to investigate blood tantalum concentrations in these patients; and (3) to report complications and mechanisms of failure related to this procedure at mid-term follow-up (mean 4.5 years).

**Methods:**

We retrospectively reviewed 29 patients (29 hips) with severe acetabular bone loss (Paprosky type III A) reconstructed using a modular tantalum TM augment in combination with a cemented cup. We evaluated the implant survival and the radiological and clinical outcomes after a mean follow-up of 4.5 years (SD 2.2; range 8.4 – 2.1 years) using patient reported outcome scores (PROMs). Blood samples were analysed regarding tantalum concentration and compared with a control group.

**Results:**

The cumulative survival rate at 4.5 years with the endpoint “revision of the acetabular component for any reason” was 96.2% (95% Confidence Interval 75.7–99.5). The PROMs improved significantly up to the latest follow-up, and radiographic data showed only one patient with signs of initial implant migration with a broken screw and a change of the position of the augment and the cup. Mean blood tantalum concentrations were significantly higher in the study group (0.16 µg/L) compared to the control group (0.002 µg/L) (*P* < 0.001).

**Conclusions:**

This study has demonstrated good mid-term (mean 4.5 years) clinical and radiological outcomes of modular tantalum TM augments in combination with a cemented cup for the reconstruction of major acetabular defects. Mean blood tantalum concentrations were increased in patients with stable tantalum implants compared to healthy controls.

## Introduction

Periacetabular bony defects remain a great challenge in total hip arthroplasty (THA) [13] and primary stable fixation and sufficient biological reconstruction of a sustainable bone stock are essential for long-term success [13]. One solution for the management of uncontained structural acetabular defects is the use of modular trabecular metal (TM) implants made of tantalum alloy. Current studies have shown that tantalum has good biocompatibility and excellent osseointegration properties [10–12, 14, 20]. Compared with conventional porous materials, these TM components have a higher coefficient of friction at the implant-bone interface, lower stiffness and higher porosity [9]. The modular augment fills the defect and enables the impaction of an uncemented porous TM press-fit cup which results in an initial primary stability. A thin layer of high-viscosity bone cement is placed between the cup and the augment to create a monolithic structure. The high three-dimensional porosity allows deep bony ingrowth in the acetabular construct with secondary biologic fixation [15]. While many studies investigate and describe this cementless press-fit technique, as proposed by the manufacturer, there is also the possibility to use modular TM augments in combination with cemented cups. Especially in cases, where the cementless technique is not appropriate due to insufficient press-fit, the use of cemented dual mobility cups or cemented PE cups in combination with modular TM augments represents a viable treatment concept. However, there is little published data in the current literature on the clinical outcomes of this treatment option. The use of dual mobility cups is an established practice in hip replacement surgery, in order to ensure higher implant stability and a reduced risk of dislocation [5]. Dual mobility cups made of cast CoCr alloy are more rigid compared to tantalum alloy implants, which could influence the tribological characteristics at the implant-implant interface of modular components and could result in a disadvantage for the stability of the overall construct. Micro-movements between the trabecular metal and the bone cement could lead to the release of tantalum particles. In addition, the higher degree of restriction due to the constrained dual mobility cup could increase the mechanical stress at the augment, which could lead to early aseptic loosening. Potential problems and mechanical complications associated with the modularity of tantalum augments such as early aseptic loosening and the potential risk of tantalum wear continue to be of concern. The purpose of this study was (1) to evaluate the radiological and clinical outcome of complex acetabular reconstruction surgery with the use of modular tantalum TM augments in combination with cemented revision cups; (2) to investigate blood tantalum concentrations in these patients; and (3) to report complications and mechanisms of failure related to this procedure at mid-term follow-up (FU).

## Materials and methods

### Patient selection and study cohort

We retrospectively reviewed data from a consecutive cohort of 29 patients with severe acetabular defects (Paprosky Type 3A) [16] who had undergone complex acetabular reconstruction using a modular tantalum TM augment (ZimmerBiomet, Warsaw, IN, USA) in combination with a cemented dual-mobility cup (AVANTAGE®, ZimmerBiomet, Warsaw, IN, USA) or a cemented Mueller low-profile polyethylene cup (ZimmerBiomet, Warsaw, IN, USA) between January 2011 to December 2020 with a minimum two year follow-up. The study was approved by the local ethics committee (S-122/2021) and all patients signed the informed consent form.

### Clinical and radiographic FU

Clinical evaluation included the Harris-Hip-Score (HHS), the UCLA Activity-Score (UCLA) and the SF-12 Score [19]. Acetabular revision was defined as any replacement of one or more of the acetabular components (the tantalum TM augment, screws, the cup or the inlay).

Radiological evaluation was conducted on standard a. p. and lateral radiographs of the hip, with assessment of radiolucencies, osteolysis or migration of the implant components up to the latest FU. Loosening of the augment/cup construct was defined if (1) > 3 mm migration of the augment/cup construct compared with the early postoperative radiograph occurred, (2) a progressive radiolucent line at the augment-bone interface, (3) radiolucent lines around all screws, or (4) screw fracture could be detected on the x-ray. The radiographs were assessed by two independent orthopaedic surgeons specialized in THA (D.S. and T.R.).

### Blood examination

A whole blood sample (7.5 ml) was taken at the FU examination from each patient as well as from 15 patients without any metal implants who served as control group. The collected blood samples were kept frozen in polypropylene tubes with sodium heparin and sent to an accredited external laboratory (Geological Institute, University Heidelberg) and analysed regarding tantalum concentration using inductively coupled plasma mass spectrometry [17]. The results of the control group have already been published as part of another study [18].

### Statistical analysis

Statistical evaluation was conducted using SPSS® Version 26.0 (IBM SPSS Statistics, IBM, Armonk, NY, USA) with a significance level set at *p* < 0.05. For descriptive data analysis demographic data were tabulated and stated as mean values with ranges and standard deviation (SD). For comparison of pre- and postoperative functional scores, the paired t-test was used. Kaplan–Meier survivorship analysis was performed with revision of the acetabular component for any reason as the end point.

## Results

### Patient cohort

**Fig. 1:**
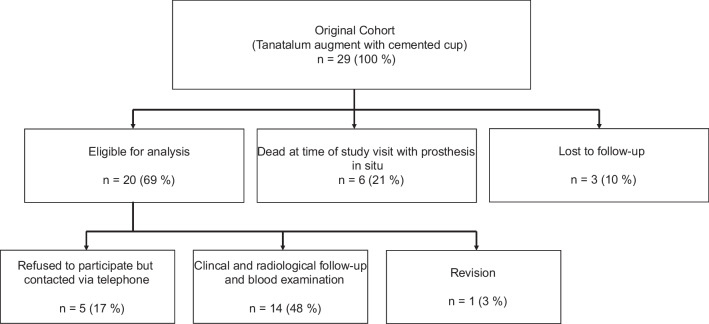
Clinical trial profile and patient flowchart

Figure 1 shows the clinical trial profile and patient flowchart. From the original cohort (*n* = 29 hips in 29 patients), three patients (10.0%) were lost to FU (address unknown / foreign country). Six patients (21.0%) had died from unrelated causes with the implant in situ. In summary, 20 patients (69%) were eligible for analysis. The mean FU of this cohort was 4.5 years (SD 2.2; range 8.4 – 2.1 years). Five patients (17.0%) refused to participate in the study, but all of these patients reported absence of a previous revision surgery. One patient underwent acetabular revision surgery. Complete clinical and radiological follow-up data were available in 14 patients (48.0%) at a mean FU of 4.1 years (SD 2.1; range 2.0 – 8.0 years). Blood samples of these 14 patients were analysed regarding blood concentrations of tantalum. Out of these 14 patients, there were seven male and seven female patients. The mean age of the patients was 72.8 years (range 51–91). The mean BMI was 27.7 kg/m2 (range 21.0–38.9).

The indication for the operation was revision THA because of aseptic loosening of the cup in four cases and two-stage revision THA for infection in four cases. The indication for primary total hip arthroplasty was severe osteoarthritis with advanced acetabular bone loss in four cases, aseptic osteonecrosis in 1 case and tumorous destruction (metastatic prostate carcinoma) in one case.

In 11 cases, a tantalum TM augment was used to fill a superior bone void and to restore the anatomical acetabular centre of rotation. In three cases a tantalum TM buttress augment was used.

**Fig. 2:**
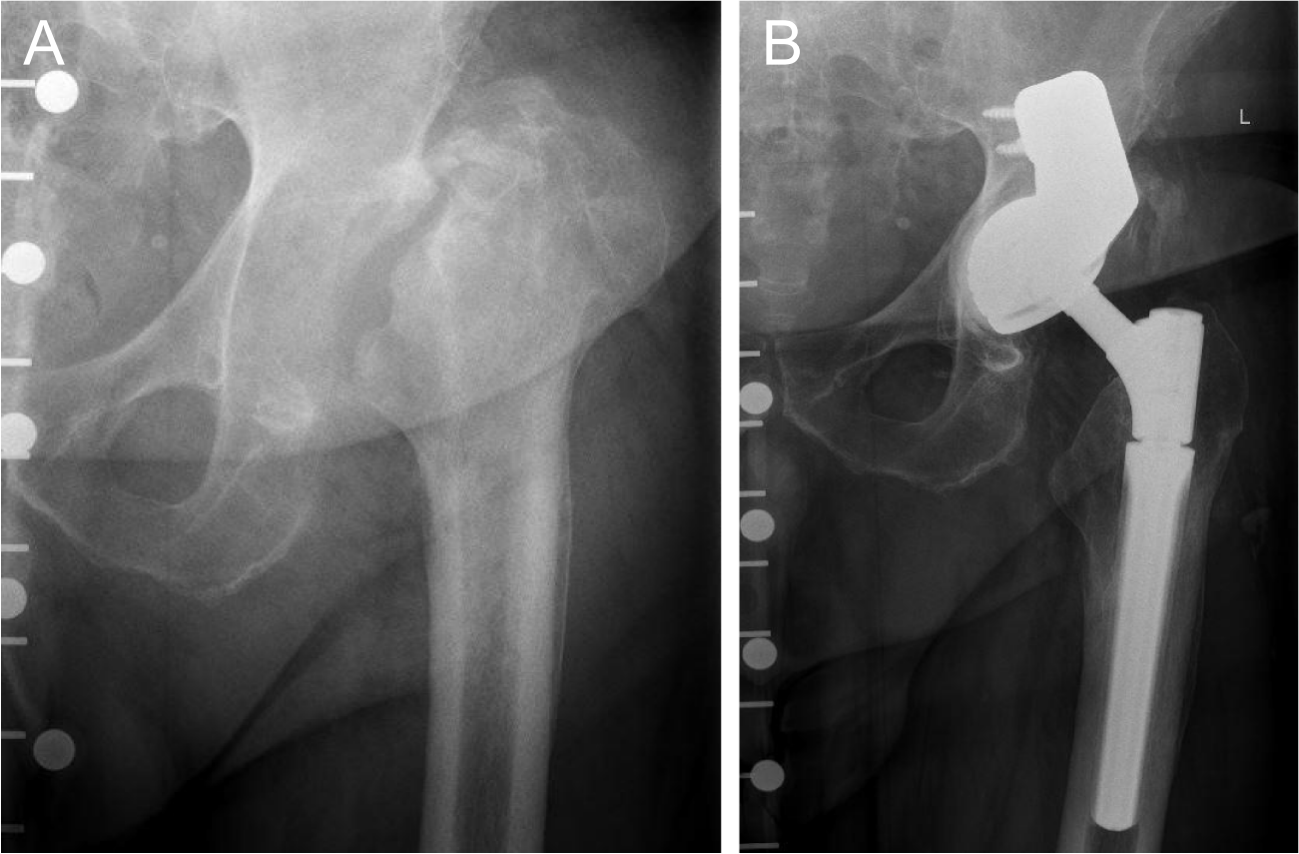
**A** Preoperative anteroposterior radiograph of the hip of a 56-year-old patient with severe acetabular bone loss including severe posterior acetabular rim defect due to infection and two-stage revision THA. **B** Anteroposterior radiograph of the same patient 4.5 years after surgery using a modular tantalum TM buttress augment and a cemented dual mobility cup. There are no signs of loosening of one of the components

**Fig. 3:**
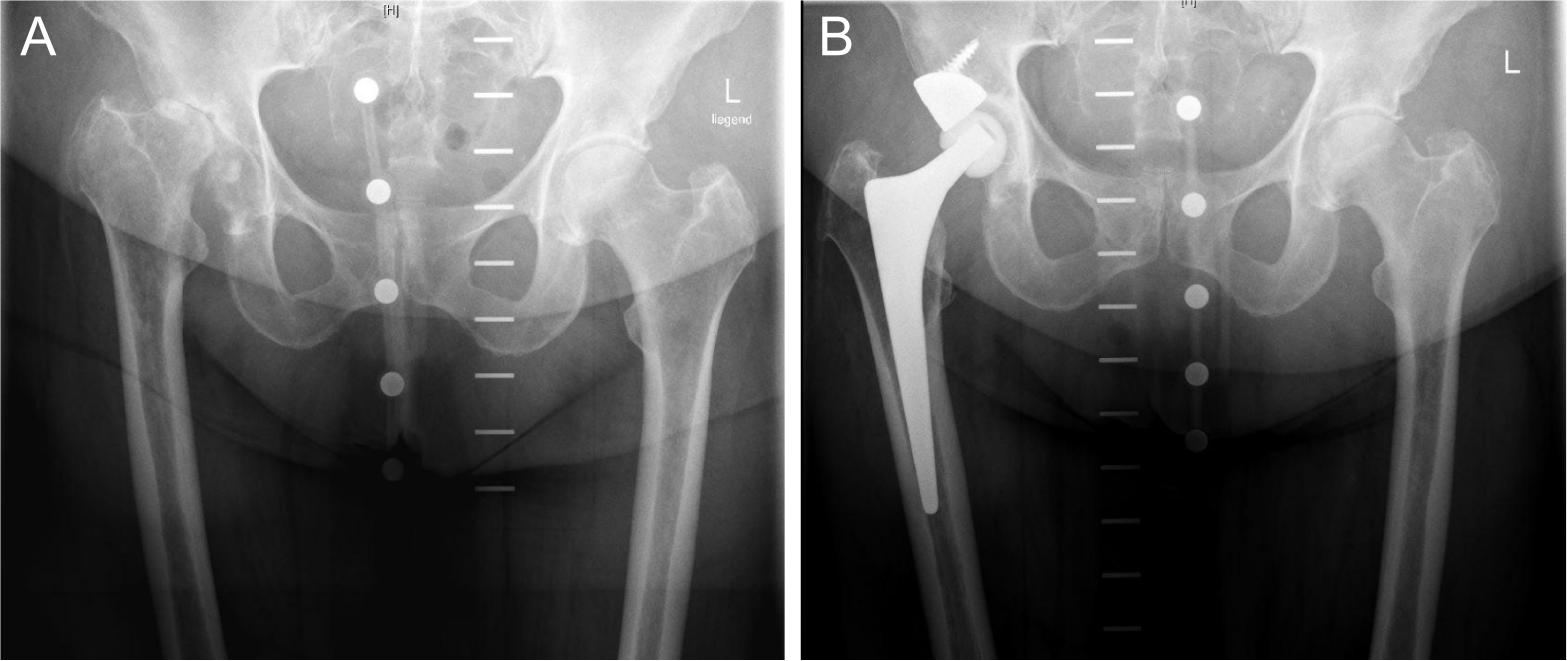
**A** Preoperative anteroposterior radiograph of the hip of a 68-year-old patient with severe osteoarthritis with advanced acetabular bone loss. **B** Anteroposterior radiograph of the same patient 6 years after surgery using a modular tantalum TM augment and a cemented PE cup. There are no signs of loosening of one of the components

In 11 cases, a dual-mobility cup was used (see Fig. 2) and in three cases (all primary total hip arthroplasty) a cemented polyethylene cup (see Fig. 3) was used.

### Survival analysis

The cumulative survival rate at 4.5 years with the endpoint “revision of the acetabular component for any reason” was 96.2% (95% Confidence Interval 75.7–99.5). At the most recent FU one patient of the study cohort had undergone revision surgery of the augment and the cup due to a re-infection. The original indication for the operation was two-stage revision of the THA due to a prosthesis infection. In this case, the stable implants had to be completely removed three months after revision surgery due to a persistent infection after an unsuccessful DAIR procedure with replacement of the mobile components five weeks postoperatively.

### Patient reported outcome measures and radiographic evaluation

**Fig. 4:**
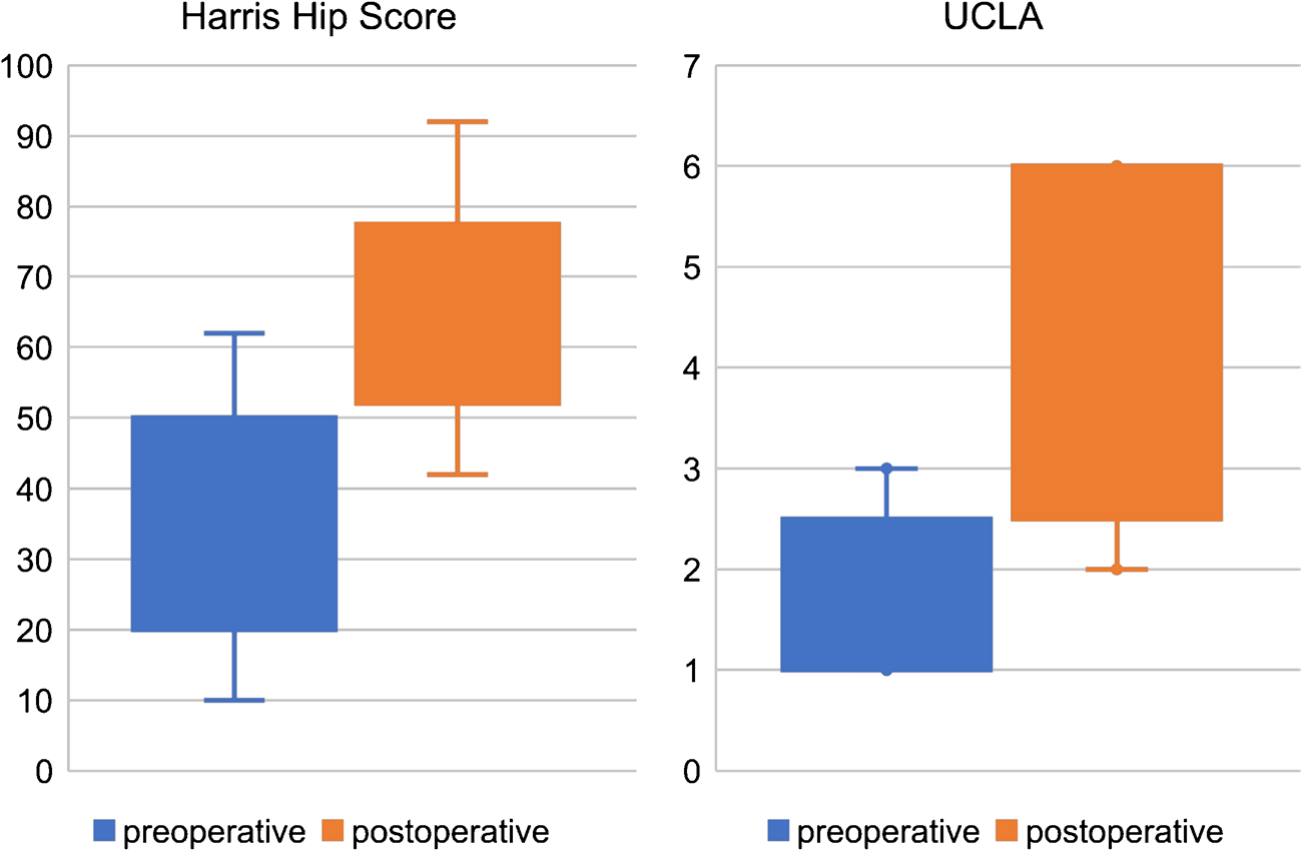
Comparison of Harris Hip Score and UCLA Score before surgery and at last follow-up, represented in Box- Whisker-Plots. The box marks the interquartile range, the band inside the box indicates the median, whiskers indicating minimum and maximum data

Mean HHS improved from 36.1 (SD 16.0; 10.0–62.0) points preoperatively to 66.3 (SD 15.5; 42.0–92.0) points at the last FU (*p* < 0.001). The UCLA Score improved from a mean preoperative score of 1.9 (SD 0.7; 1.0–3.0) points to 3.9 (SD 1.6; 2.0–6.0) points (*p* < 0.001) (see Fig. 4). Mean postoperative PCS-12 was 32.2 (SD 7.0; 20.7–39.5) points and MCS-12 was 42.6 (SD 9.7; 19.8–38.8) points.

**Fig. 5:**
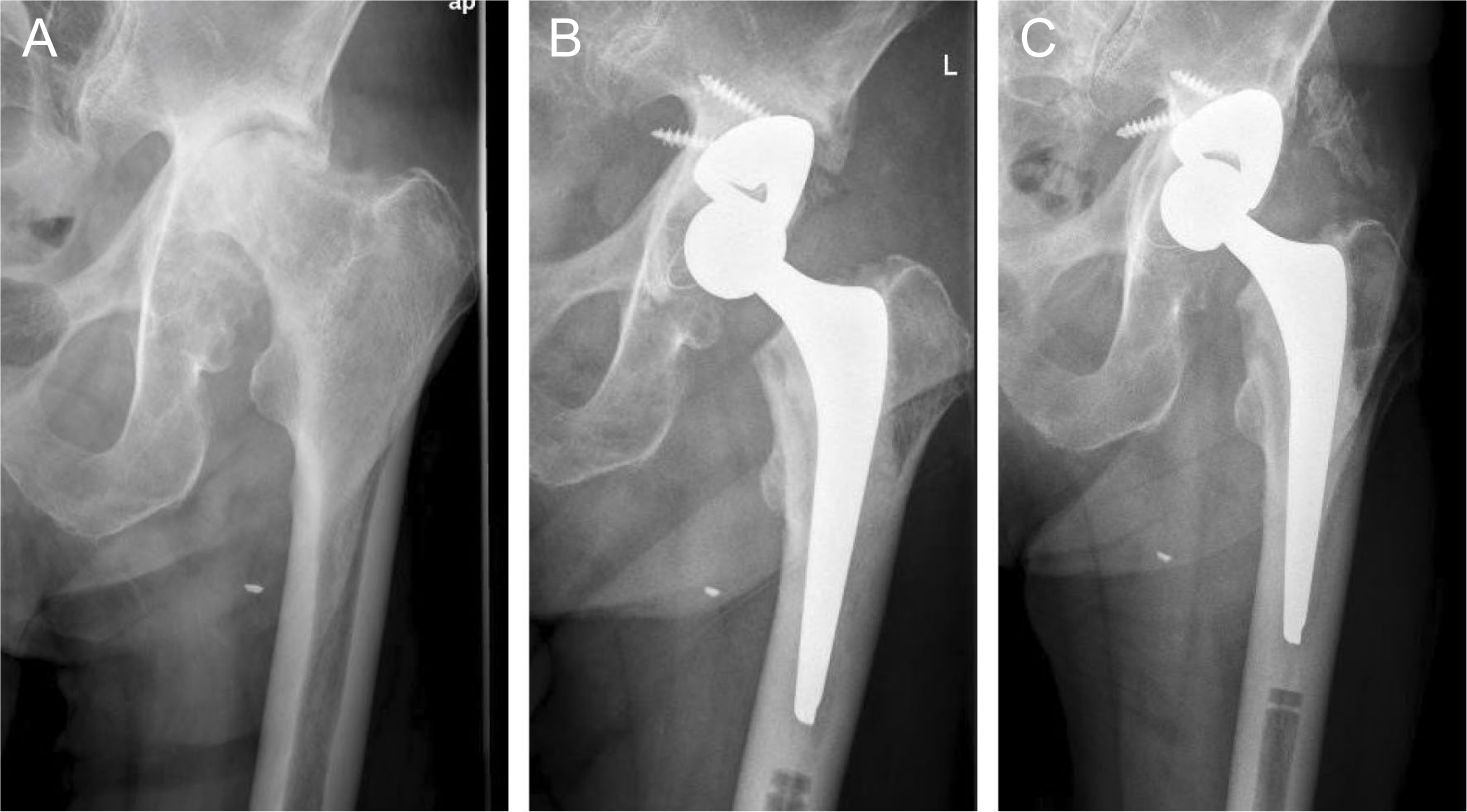
**A** Preoperative anteroposterior radiograph of the hip of a 91-year-old patient with severe osteoarthritis with advanced acetabular bone loss. **B** Anteroposterior radiograph of the same patient 3 month postoperatively, with a tantalum TM augment in combination with a cemented polyethylene cup. **C** Anteroposterior radiograph of the same patient 3.1 years postoperatively, with signs of loosening and dislocation of the tantalum augment with broken screw with unchanged cup position

At the last FU, one patient (see Fig. 5) showed signs of initial implant migration with a broken screw and a change of the position of the augment and the cup. We assume secondary osseointegration of the augment with stability of the augment/cup-construct, as no further migration between the first (1 year postoperatively) and the latest FU (3 years postoperatively) has occurred. In addition, the patient confirms absence of symptoms. In the other cases no signs of loosening or implant migration of neither the augment nor the acetabular cup was observed. Two patients developed Brooker grade 1 heterotopic ossification, two patients grade 2, and two patients grade 3 [3].

### Blood examination

Blood samples from the patients demonstrated a mean tantalum concentration of 0.16 µg/l (SD 0.3, range 0.02 – 0.87 µg/l). Tantalum concentrations of the study group were statistically significantly higher when compared to the control group (mean 0.002 µg/l; SD 0.001, range 0.0002 – 0.002 µg/l) (*p* < 0.001) [18].

## Discussion

The management of severe acetabular defects is challenging [6] and adequate primary stability of the acetabular construct is crucial for the long-term survival of the implant [2]. Uncontained, larger defects with insufficient acetabular bone support often necessitate the use of modular porous coated metal augments in combination with a cementless press-fit shell [7]. Currently, the most frequently used cementless revision cups with a porous metal surface consist either of tantalum or titanium metal alloys. The press-fit implantation provides a stable mechanical interface between the implant and the host bone in the short term (primary stability), and the porous structure facilitates osseointegration for secondary stability and long-term fixation [6, 7]. Tantalum in the form of TM is currently one of the more frequently used porous implants [2] and can be used to treat extensive acetabular defects [15] as well as neoplastic periacetabular lesions [2]. However, press-fit anchoring of a cup is sometimes not possible, for example due to reduced bone quality or cavitary acetabular defects. In these cases, the use of a cemented cup in combination with a modular TM augment can be an option. However, there are only a few studies in the literature that have investigated the clinical outcome of this treatment strategy so far. The aim of this study was to evaluate the clinical and radiological results of modular tantalum TM augments in combination with a cemented cup for reconstruction of acetabular bone defects in THA and to investigate the systemic blood tantalum exposure in these patients.

In our cohort, there was a cumulative survival rate of 96.2% for the endpoint “revision of the acetabular component for any reason” at a mean FU of 4.5 years. At the most recent FU only one patient of the study cohort has had revision surgery of the augment and the cup due to infection. There was no case of aseptic loosening requiring revision surgery of the cup or augment. At the latest FU, one patient demonstrated signs of initial implant migration with a broken screw and a change of the position of the augment and the cup without clinical symptoms. We assume secondary osseointegration of the augment with stability of the augment/cup-construct, as no further migration between the first (1 year postoperatively) and the latest FU (3 years postoperatively) has occurred. Previous studies have shown good clinical and radiological results of modular tantalum TM augments in combination with cementless TM revision cups for reconstruction of acetabular bone defects in THA. Alqwbani et al. reported excellent clinical results in 48 hips at a mean FU of 6.25 years with an overall survivorship of the acetabular component of 100% and full osseointegration in all cases [1]. Comparable results were published by Flecher et al. [9], who reported a global survivorship of 92.3% at 5.3 years in 51 hips. Eachempati et al. [8] reported no implant failure and a survivorship of 100% in 41 hips at a mean FU of 3.3 years. Our data showed significant improvement in HHS and UCLA and satisfactory values for the VR-12 test. Other studies with comparable study design have also demonstrated excellent clinical outcomes for the cementless technique [1, 8]. For a complete biological reconstruction, a cementless restoration should be aimed for. The results of our study confirm that in situations where the press-fit technique is not possible for anatomical reasons modular tantalum TM augments in combination with a cemented revision cup provide good initial stability with a high rate of secondary osseointegration of the augment and a stable augment-cup-construct with satisfactory clinical results at a mid-term FU.

Micromotion may potentially occur between the cement and the augment or, in case of a poorly cemented dual-mobility cup, between the metallic shell and the augment which can lead to metal wear. The resulting friction and mechanical wear can lead to the release of tantalum particles into the periarticular tissue and might cause early aseptic loosening due to implant debonding. In our cohort, mean blood tantalum concentrations at mid-term FU were increased (0.16 µg/l) in patients with stable implants compared to our control group (0.002 µg/l) [18]. To our knowledge, a reference value for serum tantalum concentrations has only been described for a healthy population not exposed to tantalum implants [4, 17] and is specified up to 0.01 µg/l [17]. To our knowledge, no study has investigated blood tantalum concentrations in patients treated with modular tantalum TM augments in combination with a cemented cup. Bruggemann et al. [4] reported elevated blood tantalum concentrations in patients treated with a cementless tantalum cup in primary THA (0.051 µg/l) and in patients who underwent revision surgery with a cementless tantalum revision shell (0.091 µg/l) without the use of tantalum augments after four years. Our results demonstrated higher levels of tantalum (0.16 µg/l) in the blood after 4.5 years. This could be a consequence of the higher implant volume or increased friction at the cement-augment interface. In general, tantalum is considered to be highly biocompatible [4], but the local and systemic effects of highly elevated tantalum levels have not yet been sufficiently investigated and should be the subject of further research.

This study has some limitations that have to be acknowledged. One the one hand, the study is limited due to the retrospective study design and the relatively small sample size of the cohort. This is due to the fact that the indications for using modular tantalum TM augments in combination with a cemented cup are rare and only 29 of these procedures were performed over a span of nine years at our institution. Secondly, the FU period is relatively short and long-term FU data of these patients are not yet available. However, to our knowledge, this is the first study to evaluate both the radiological and clinical outcomes of complex acetabular reconstruction using modular tantalum augments in combination with cemented revision cups and additionally investigated the blood tantalum concentrations in these patients.

## Conclusion

In this study, favourable mid-term (mean 4.5 years) clinical and radiological outcomes of modular tantalum TM augments in combination with a cemented cup for reconstructing major acetabular defects were observed. Patients with stable tantalum implants exhibited higher mean blood tantalum concentrations compared to healthy controls. Although the overall tantalum levels were relatively low, the potential local or systemic effects of an increased tantalum exposure warrant further investigation. Further long-term follow-up data are necessary to longitudinally assess the clinical results and revision-free survival of these modular cemented cup implants.

## Data Availability

The datasets used and/or analysed during the current study are available from the corresponding author on reasonable request.

## References

[1] Alqwbani M, Wang Z, Wang Q et al (2022) Porous tantalum shell and augment for acetabular defect reconstruction in revision total hip arthroplasty: a mid-term follow-up study. Int Orthop 46:1515–1520 35224670 10.1007/s00264-022-05353-w

[2] Beckmann NA, Bitsch RG, Schonhoff M et al (2020) Comparison of the primary stability of porous tantalum and titanium acetabular revision constructs. Materials (Basel) 13(7):1783 32290103 10.3390/ma13071783 PMC7179011

[3] Brooker AF, Bowerman JW, Robinson RA et al (1973) Ectopic ossification following total hip replacement. Incidence and a method of classification. J Bone Joint Surg Am 55:1629–1632 4217797

[4] Bruggemann A, Mallmin H, Bengtsson M et al (2020) Safety of use of tantalum in total hip arthroplasty. J Bone Joint Surg Am 102:368–374 31895169 10.2106/JBJS.19.00366

[5] Ciolli G, Mesnard G, Deroche E et al (2022) Is cemented dual-mobility cup a reliable option in primary and revision total hip arthroplasty: a systematic review. J Pers Med 13(1):81 36675742 10.3390/jpm13010081 PMC9867154

[6] Ciriviri J, Nestorovski Z, Talevski D et al (2019) Treatment of acetabular defects with porous metal augments in revision hip surgery. Pril (Makedon Akad Nauk Umet Odd Med Nauki) 40:33–39 31605583 10.2478/prilozi-2019-0012

[7] Dwivedi C, Gokhale S, Khim HG et al (2017) Acetabular defect reconstruction with trabecular metal augments: study with minimum one-year follow-up. Hip Pelvis 29:168–175 28955682 10.5371/hp.2017.29.3.168 PMC5612976

[8] Eachempati KK, Malhotra R, Pichai S et al (2018) Results of trabecular metal augments in Paprosky IIIA and IIIB defects: A multicentre study. Bone Joint J 100-B:903–908 29954197 10.1302/0301-620X.100B7.BJJ-2017-1604.R1

[9] Flecher X, Appy B, Parratte S et al (2017) Use of porous tantalum components in Paprosky two and three acetabular revision. A minimum five-year follow-up of fifty one hips. Int Orthop 41:911–916 27766385 10.1007/s00264-016-3312-2

[10] Han Q, Wang C, Chen H et al (2019) Porous Tantalum and Titanium in Orthopedics: A Review. ACS Biomater Sci Eng 5:5798–5824 33405672 10.1021/acsbiomaterials.9b00493

[11] Huang G, Pan ST, Qiu JX (2021) The clinical application of porous tantalum and its new development for bone tissue engineering. Materials (Basel) 14(10):2647 34070153 10.3390/ma14102647 PMC8158527

[12] Kato H, Nakamura T, Nishiguchi S et al (2000) Bonding of alkali- and heat-treated tantalum implants to bone. J Biomed Mater Res 53:28–35 10634949 10.1002/(sici)1097-4636(2000)53:1<28::aid-jbm4>3.0.co;2-f

[13] Koob S, Scheidt S, Randau TM et al (2017) Biological downsizing: Acetabular defect reconstruction in revision total hip arthroplasty. Orthopade 46:158–167 28074234 10.1007/s00132-016-3379-x

[14] Levine BR, Sporer S, Poggie RA et al (2006) Experimental and clinical performance of porous tantalum in orthopedic surgery. Biomaterials 27:4671–4681 16737737 10.1016/j.biomaterials.2006.04.041

[15] O’neill CJ, Creedon SB, Brennan SA et al (2018) Acetabular revision using trabecular metal augments for Paprosky type 3 defects. J Arthroplasty 33:823–828 29217393 10.1016/j.arth.2017.10.031

[16] Paprosky WG, Perona PG, Lawrence JM (1994) Acetabular defect classification and surgical reconstruction in revision arthroplasty. A 6-year follow-up evaluation. J Arthroplasty 9:33–44 8163974 10.1016/0883-5403(94)90135-x

[17] Rodushkin I, Engstrom E, Stenberg A et al (2004) Determination of low-abundance elements at ultra-trace levels in urine and serum by inductively coupled plasma-sector field mass spectrometry. Anal Bioanal Chem 380:247–257 15322793 10.1007/s00216-004-2742-7

[18] Spranz D, Muller LM, Trefzer R et al (2024) Elevated blood tantalum concentrations in patients following reconstruction of severe acetabular defects in total hip arthroplasty using modular tantalum augments in combination with uncemented tantalum cups. J Arthroplasty. 10.1016/j.arth.2024.05.068 38823519 10.1016/j.arth.2024.05.068

[19] Ware J Jr, Kosinski M, Keller SD (1996) A 12-item short-form health survey: construction of scales and preliminary tests of reliability and validity. Med Care 34:220–233 8628042 10.1097/00005650-199603000-00003

[20] Ying J, Cheng L, Li J et al (2023) Treatment of acetabular bone defect in revision of total hip arthroplasty using 3D printed tantalum acetabular augment. Orthop Surg 15:1264–1271 36896785 10.1111/os.13691 PMC10157706

